# Hi-*JAKi*-ng Synovial Fibroblasts in Inflammatory Arthritis With JAK Inhibitors

**DOI:** 10.3389/fmed.2020.00124

**Published:** 2020-05-05

**Authors:** Blaž Burja, Tonja Mertelj, Mojca Frank-Bertoncelj

**Affiliations:** ^1^Center of Experimental Rheumatology, University Hospital Zurich, Schlieren, Switzerland; ^2^Department of Rheumatology, University Medical Centre Ljubljana, Ljubljana, Slovenia; ^3^Faculty of Medicine, University of Ljubljana, Ljubljana, Slovenia

**Keywords:** synovial fibroblasts, fibroblast-like synoviocytes, JAK inhibitors, STATs, rheumatoid arthritis

## Abstract

The Janus kinase (JAK)—Signal transducer and activator of transcription (STAT) pathway is one of the central signaling hubs in inflammatory, immune and cancer cells. Inhibiting the JAK-STAT pathway with JAK inhibitors (jakinibs) constitutes an important therapeutic strategy in cancer and chronic inflammatory diseases like rheumatoid arthritis (RA). FDA has approved different jakinibs for the treatment of RA, including tofacitinib, baricitinib and upadacitinib, and several jakinibs are being tested in clinical trials. Here, we reviewed published studies of jakinib effects on resolving synovial pathology in inflammatory arthritis. We discussed the results of jakinibs on structural joint damage in clinical trials and explored the effects of jakinibs across different *in vitro, ex vivo*, and *in vivo* synovial experimental models. We delved rigorously into experimental designs of *in vitro* fibroblast studies, deconvoluted jakinib efficacy in synovial fibroblasts across diverse experimental conditions and discussed their translatability *in vivo*. Synovial fibroblasts can readily activate the JAK-STAT signaling pathway in response to cytokine stimulation. We highlighted rather limited effects of jakinibs on the *in vitro* cultured synovial fibroblasts and inferred that direct and indirect (immune cell-dependent) actions of jakinibs are required to curb the fibroblast pathology *in vivo*. These actions have not been mimicked optimally in current *in vitro* experimental designs, where inflammatory stimuli do not naturally clear out with treatment as they do *in vivo*. While summarizing the broad knowledge of synovial jakinib effects, our review uniquely challenges future study designs to better mimick the jakinib actions in broader cell communities, as occurring *in vivo* in the inflamed synovium. This can deepen our understanding of collective synovial activities of jakinibs and their therapeutic limitations, thereby fostering jakinib development in arthritis.

## JAK-Stat Signaling Pathway and JAK Inhibitors

The Janus kinase (JAK)—Signal transducer and activator of transcription (STAT) signaling pathway is a central signaling hub in human immune responses and cancer. This pathway transmits signals from a plethora of growth factors [e.g., epidermal growth factor (EGF), platelet-derived growth factor (PDGF)] and cytokines [e.g., interleukin-6 (IL-6), type I and II interferons (IFN), IL-7, IL-15]. Cytokines and growth factors, their membrane-bound/soluble receptors and downstream signaling events in the JAK-STAT pathway have been extensively covered elsewhere (1–3). Briefly, the canonical JAK-STAT signaling pathway is initiated upon receptor ligation (with a cytokine or a growth factor) and dimerization followed by auto-phosphorylation and activation of the receptor-associated JAKs. Auto-phosphorylated JAKs phosphorylate cytoplasmic receptor tails, which serve as docking sites for cytoplasmic STATs. Upon phosphorylation by JAKs, STAT dimers translocate to the nucleus, bind to specific promoter sequences, and induce gene transcription. In mammals, different JAKs [JAK1-3, Tyrosine kinase 2 (TYK2)] associate with distinct receptors and preferentially phosphorylate different STATs. There are seven STATs encoded in the human genome, including STAT1-4, STAT5a, STAT5b, and STAT6. STATs form specific homo- or hetero-dimers, thereby inducing different effector genes. Individual STATs can be activated with multiple ligands (heterogeneous signaling) and individual cytokines can activate multiple STATs, albeit exhibiting a preference for a specific subset of STATs. IL-6 (4) and oncostatin M (OSM) (5) prototypically activate STAT3, whereas type I interferons primarily activate STAT1 and STAT2 with a weaker activation of STAT4 (6). IFNγ potently activates STAT1 with a weaker activation of STAT3 (7).

The activation of the JAK-STAT pathway is tightly controlled at multiple steps of the signaling cascade. Diverse phosphatases, including SHP1, SHP2, and DUSP2 remove phosphate groups from phosphorylated receptors, JAKs and STATs (8, 9). The protein inhibitors of activated STAT (PIAS) inhibit STAT binding to target DNA sequences, control subcellular location of STATs and facilitate post-translational modifications of STATs (10). Additionally, suppressors of cytokine signaling (SOCS) competitively inhibit binding of STATs to cytokine receptors but act also as ubiquitin ligases, targeting the components of the JAK-STAT pathway for proteosomal degradation (11). The human genome encodes eight different SOCS proteins, including SOCS1-7 and cytokine inducible regulator (CIS) proteins (12). STATs positively regulate transcription of *SOCS* genes, which creates a negative feedback loop in the JAK-STAT signaling cascade, thereby enabling the fine-tuning of the pathway outputs (13).

JAK-STAT pathway has been intensively studied in diverse mouse models [as reviewed in (14, 15)] and human studies (16). These studies showed that exaggerated or protracted JAK-STAT signaling leads to aberrant development of hematopoietic stem cells, hematological malignancies, and immunodeficiency syndromes. Specifically, loss-of-function mutations in the JAK-STAT pathway, e.g., in *JAK3* gene, led to immunodeficiency disorders (17, 18), whereas gain-of-function mutations, e.g., in *JAK2* gene, caused human lymphoproliferative diseases (19–21). Additionally, the JAK-STAT pathway has been closely linked with antiviral (22, 23) inflammatory and autoimmune responses in a variety of human tissues and diseases (24–26).

The fundamental position of the JAK-STAT pathway at the crossroad of inflammatory, autoimmune and cancer pathologies has driven the discovery and therapeutic success of JAK inhibiting drugs (jakinibs). In November 2011, ruxolitinib, the potent inhibitor of JAK1 and JAK2, became the first approved jakinib by the US Food and Drug Administration (FDA). Ruxolitinib was authorized for the treatment of intermediate and high-risk myelofibrosis and polycythemia vera in patients with inadequate response or intolerance for hydroxyurea (27). In 2012, tofacitinib, the pan-JAK inhibitor that primarily inhibits JAK1 and JAK3, and to a lesser extent JAK2, followed as the second FDA-approved jakinib, and the first jakinib approved for the treatment of RA (28) (Table 1). Since then, several other jakinibs have entered clinical trials in patients with inflammatory arthritis and other inflammatory diseases (e.g., ulcerative colitis, psoriasis), as reviewed in Winthrop (29) and O'Shea and Gadina (30). Tofacitinib has been FDA-approved for psoriatic arthritis (PsA), whereas baricitinib (31) (the JAK1 and JAK2 inhibitor) and upadacitinib (32) (the selective JAK1 inhibitor) have been FDA-approved for RA (Table 1). Increased selectivity of the second generation jakinibs like upadacitinib toward inhibiting a single JAK can be beneficial, decreasing the possibility of jakinib-driven side effects.

**Table 1:** FDA-approved jakinibs for the treatment of autoimmune inflammatory arthritis.

**Jakinib**	**Approved indications in patients with inflammatory arthritis**	**Approval status**
Tofacitinib JAK1,3 less JAK2 selective 5 mg bi-daily	Adult patients with moderately-to-severely active RA who inadequately respond or are intolerant to MTX. Tofacitinib may be used as monotherapy or in combination with MTX or other non-biologic DMARDs Adult patients with active PsA who inadequately respond/are intolerant to MTX or other DMARDs	FDA approved November 2012 FDA approved November 2016
Baricitinib JAK1,2 selective 2–4 mg once daily	Moderately-to-severely active RA with inadequate response to TNF inhibitors, as monotherapy or in combination with non-biologic DMARDs	FDA approved June 2018
Upadacitinib JAK1 selective 15–30 mg once daily	Moderately to severely active RA with inadequate response/intolerance for MTX	FDA approved August 2019
Peficitinib JAK pan-inhibitor 150 mg once daily	RA (including prevention of structural joint damage) in patients with inadequate response to conventional DMARDs	Approved in Japan March 2019

*JAK1-3, Janus kinase 1-3; MTX, methotrexate; DMARDs, disease modifying antirheumatic drugs; FDA, US Food and Drug Administration; RA, Rheumatoid arthritis; PsA, Psoriatic arthritis*.

## The Effects of Jakinibs on Structural Joint Damage and Synovitis in RA Clinical Trials

The effects of jakinibs in inflammatory arthritis have been tested in 100 registered clinical trials, of which 52 have been completed. In these trials different jakinibs have been evaluated, including tofacitinib (*n* = 48 clinical trials), baricitinib (*n* = 17), upadacitinib (*n* = 16), filgotinib (*n* = 11), and peficitinib (*n* = 9) in combination with other disease modifying antirheumatic drugs (DMARDs) or as monotherapy.

Here we reviewed the currently registered clinical trials on jakinibs in RA (clinicaltrials.gov database), in which structural joint changes or synovitis were assessed as an outcome using different imaging modalities. In the search, we used the following keywords: tofacitinib, CP-690550, tasocitinib, CKD374, baricitinib, INCB028050, LY3009104, upadacitinib, peficitinib, ASP015K, filgotinib, GLPG0634, rheumatoid arthritis. We identified four trials (Table 2), investigating the effects of tofacitinib on structural joint damage in patients with RA. Radiographic joint changes at baseline and during the study were assessed using X-ray, ultrasound, or magnetic resonance imaging (MRI).

**Table 2:** Clinical trials in which jakinib effects were assessed on structural joint changes and synovitis.

**Study**	**Treatment arms (Background therapy)**	**Number of participants, study durationImaging modalityStructural endpoints**
Oral Scan (NCT00847613) Interventional, double-blind, parallel-group, placebo-controlled, phase 3	tofacitinib 5 mg BID tofacitinib 10 mg BID Placebo to tofacitinib 5 mg Placebo to tofacitinib 10 mg (MTX)	797 participants, 98.7% with structural data, 24 months X-ray mTSS at month 6, 12, and 24 Change from baseline in mTSS at month 6
Oral Start (NCT01039688) Interventional, phase 3	tofacitinib 5 mg BID tofacitinib 10 mg BID MTX	956 participants (93.0% with structural data), 6 months X-ray mTSS at month 6 Changes from baseline in mTSS at month 6
Effects of tofacitinib on magnetic resonance imaging-assessed joint structure in early RA (NCT01164579) Interventional, open-label, phase 4	tofacitinib 10 mg BID + MTX tofacitinib 10 mg BID + placebo MTX Placebo tofacitinib + MTX	109 participants, 12 months X-ray, MRI Change from Baseline to Month 1, 3, 6, 12 in OMERACT RAMRIS Synovitis, Bone Marrow Oedema, Erosions (Wrist, MCP) mTSS, erosion score, joint space narrowing at month 6, 12. Change from baseline in mTSS, erosion score, joint space narrowing at month 6, 12
Musculoskeletal ultrasound assessment of therapeutic response of tofacitinib in RA patients (NCT02321930) Interventional, open-label, phase 4	tofacitinib 5 mg BID (DMARDs/prednisone <10 mg)	37 participants, 3 months Ultrasound Baseline PDUS and GSUS, Change (week 2, month 3) in PDUS, GSUS

*BID, bi-daily; DMARDs, disease modifying antirheumatic drugs; MTX, methotrexate; PDUS, The power Doppler synovitis score; GSUS, gray scale synovial hypertrophy score; MRI, magnetic resonance imaging; OMERACT, The Outcome Measures in Rheumatology Clinical Trials; RAMRIS, Rheumatoid Arthritis Magnetic Resonance Imaging Score; mTSS, modified Total Sharp Scoring system; MCP, metacarpophalangeal; RA, rheumatoid arthritis*.

The structural joint damage in ORAL Start (NCT01039688) (33), ORAL Scan (NCT00847613) (34) and the NCT01164579 phase 2 study was assessed using the X-ray imaging and the modified Total Sharp Scoring system (mTSS, range 0–488). TSS is a sum of erosion and joint space narrowing scores on 16 joints of the hand and six joints of the foot. In ORAL Scan trial (NCT00847613), patients with RA, who had inadequate response to methotrexate (MTX) monotherapy, received tofacitinib 5 or 10 mg BID (bi-daily) on background MTX. Tofacitinib 10 mg BID significantly improved mTSS at month 6 compared to MTX only (*p* < 0.05). In the ORAL Start (NCT01039688), mean changes in mTSS at month 6 were significantly smaller in MTX-naïve patients with RA who were receiving tofacitinib 5 and 10 mg BID compared with MTX only group (*p* < 0.001). The phase 2 NCT01164579 study used MRI and x-ray imaging to evaluate the effects of tofacitinib on structural joint damage in MTX-naïve patients with an early active RA (35). Treatment of these patients with tofacitinib (10 mg BID monotherapy or combined with MTX) improved the Rheumatoid Arthritis Magnetic Resonance Imaging Score (RAMRIS) bone marrow oedema at month 6, the automated RAMRIS (RAMRIQ) synovitis score at month 3 as well as RAMRIS and RAMRIQ erosive damage scores at month 6 and 12 when compared to MTX monotherapy. Numerical changes in mTSS, joint space narrowing and erosions at month 6 and 12 as compared to baseline were small in all three patient groups.

In a pilot, open-label, phase IV study (NCT02321930), synovitis was assessed in patients with RA, who were treated with tofacitinib 5 mg BID, using ultrasound assessment of 30 joints. The power Doppler synovitis score (PDUS, on 30 joints) and the gray scale synovial hypertrophy score (GSUS, on 30 joints) showed significant improvement from baseline (*p* < 0.0001) at 12 weeks of tofacitinib therapy.

A randomized, double blind, placebo-controlled, phase II study (A3921073, NCT00976599) (36) involved patients with active RA who inadequately responded to MTX and started the treatment with tofacitinib 10 mg BID on background MTX. Synovial biopsies taken at 28 days of tofacitinib therapy showed significant decrease in the expression of matrix metalloproteinase 1 (MMP1), MMP3 and interferon-regulated genes such as C-C motif chemokine ligand 2 (CCL2), C-X-C motif chemokine (CXCL10), C-X-C chemokine 13 (CXCL13). Meanwhile, the expression of interleukin 6 (IL-6), interferon-stimulated gene 15 (ISG15), and tumor necrosis factor (TNF) mRNAs was not altered in comparison with their expression in the baseline biopsies (taken 4–10 days before starting tofacitinib). Total inflammation score and the abundances of CD3+, CD20+, and CD68+ sublining macrophages did not change with 28 days of therapy. Clinical improvement as defined by disease activity score 28 (DAS28) at 4 months of therapy (NCT00413699) correlated strongly with the change in synovial pSTAT1 and pSTAT3 amounts at day 28 vs. baseline.

Overall, these studies demonstrated that tofacitinib—in combination with MTX or as monotherapy—decreased the progression of structural damage in patients with RA. Tofacitinib led to an early reduction in synovitis and inhibited the progression of structural joint damage in patients with early active RA. Early synovial responses to tofacitinib appeared to be transcriptional and not cellular (=not altering abundancy of major cell types) (36), and early blockade of STAT phosphorylation could contribute importantly to clinical improvement at 4 months of tofacitinib therapy.

## JAKINIB Effects on Synovial Explants From Patients With Inflammatory Arthritis

Synovial explants maintain synovial architecture and cell-to-cell contacts, closely mimicking synovial cellular networks in the inflamed joints (37). Treatment of synovial explants from RA patients with tofacitinib (1,000 nM, 72 h) inhibited the production of IL-6, IL-8, IL-1β, intercellular adhesion molecule 1 (ICAM1), vascular endothelial growth factor (VEGF), tyrosine kinase with Ig and EGF (epidermal growth factor) homology domains (TIE-2) and MMP1 as well as decreased invasion and outgrowth of synovial fibroblasts from synovial explants (37). Additionally, tofacitinib (1,000 nM, 72 h) showed beneficial effects on synovial explants from PsA patients. Synovial biopsies from PsA patients exhibited increased pSTAT1 and pSTAT3 (38) and 1,000 nM tofacitinib blocked phosphorylation of STAT3 and STAT1. This was accompanied by an increase in the JAK-STAT pathway inhibitors (SOCS3, PIAS3) and a decrease in IL-6, IL-8, and monocyte chemoattractant protein 1 (MCP1), but not IL-10 nor interferon gamma-induced protein 10 (IP-10). Additionally, tofacitinib partially inhibited NF-kBp65 and decreased MMP2, MMP9 and MMP3 proteins without affecting tissue inhibitor of metalloproteinases 3 (TIMP3). Similar tofacitinib effects on pSTAT3, pSTAT1, PIAS3, and SOCS3 proteins were observed in PsA synovial fibroblasts, with concomitant attenuation of migration and invasion (48 h) of synovial fibroblasts (38). These results suggested that tofacitinib might exhibit synergistic inhibitory effects on synovial pathology in RA by directly inhibiting JAKs and upregulating the endogenous inhibitors of the JAK-STAT pathways.

## JAKINIB Effects in Synovial Fibroblasts

### Inflamed Synovial Microenvironment Activates the JAK-STAT Signaling in Synovial Fibroblasts

**Figure 1:**
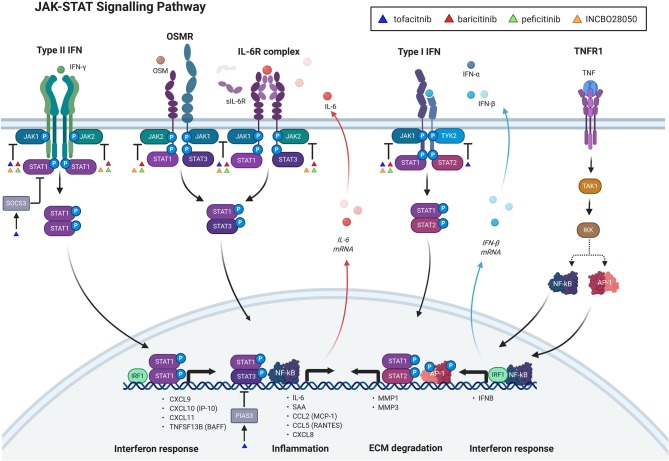
Janus kinase (JAK)-Signal transducer and activator of transcription (STAT) signaling pathway and its inhibition with jakinibs in synovial fibroblasts. The figure shows direct and indirect signaling pathways, involved in the JAK-STAT signaling and their targeting with jakinibs in synovial fibroblasts. Only pathways with mechanistic evidence and jakinibs with experimental evidence in synovial fibroblasts are included in the figure. Upadacitinib has not yet been experimentally explored in synovial fibroblasts and is therefore not shown in the figure. Direct signaling through the JAK-STAT pathway can be induced with type I and type II interferons (IFN) and interleukin-6 (IL-6) family cytokines [oncostatin M (OSM), IL-6] upon ligating their respective receptors. This is followed by auto-phosphorylation of distinct JAKs that phosphorylate specific subsets of STATs. Tumor necrosis factor (TNF) indirectly activates the JAK-STAT pathway by increasing the release of IL-6 and IFN beta (IFNβ) from synovial fibroblasts with subsequent autocrine activation of IL-6 family and IFN Type I receptor complexes, respectively. sIL-6R: soluble IL-6 receptor; TNFR1: TNF receptor 1; IFNα: interferon alpha; NF-κB: nuclear factor-kappa B; AP-1: activator protein 1; TAK1: transforming growth factor beta-activated kinase 1; IKK: inhibitory kappa B kinase; SOCS3: suppressor of cytokine signaling 3; IRF1: interferon response factor 1; CXCL8-11: chemokine (C-X-C motif) ligand 8–11; IP-10: IFN-γ-inducible protein 10; TNFSF13B: tumor necrosis factor ligand superfamily member 13B, also known as BAFF, BAFF: B-cell activating factor; PIAS3: protein inhibitor of activated STAT3; CCL2: chemokine (C-C motif) ligand 2; MCP-1: monocyte chemoattractant protein 1; SAA: serum amyloid A; MMP 1,3: matrix metalloproteinase 1,3; ECM: extracellular matrix; INCBO28050: baricitinib. The figure was created with BioRender.

Synovial fibroblasts, stimulated with TNF, IL-1α, or Poly(I:C) secreted a pattern of cytokines which correlated significantly with the cytokine profile in synovial fluid from RA patients (39). This suggested that synovial fibroblasts have a substantial role in shaping the inflamed microenvironment in RA joints (39). Specifically, the top six induced cytokines, the so called six cytokine set [IL-6, regulated upon activation, normal T cell expressed and presumably secreted (RANTES), growth regulated oncogene-alpha (GRO-α), MCP-1, IL-8, and IP-10] were among the 25% most abundant cytokines in RA synovial fluid (out of 48 measured) (39). Most of these molecules, in particularly IL-6, RANTES, MCP-1, and IP-10 closely associate with the JAK-STAT signaling in synovial fibroblasts (Figure 1). Synovial fibroblasts activated the JAK-STAT signaling pathways upon exposure to RA synovial fluid (40, 41), immune/inflammatory cell-derived cytokines [OSM (42), TNF, IFNγ] as well as autocrinally-produced IL-6 and type I interferons (Figure 1).

This indicates that synovial fibroblasts represent a prominent synovial source of the JAK-STAT pathway-associated cytokines and that a complex JAK-STAT pathway-guided crosstalk between synovial fibroblasts and immune cells can aggravate synovial pathology in RA. Thus, the efficacy of jakinibs in RA could reflect their concomitant actions on individual synovial cell types as well as their networks in RA. Jakinib actions on immune cells in inflammatory arthritis have been extensively studied and reviewed elsewhere (43). Here we focused on reviewing jakinib actions on synovial fibroblasts, exposed to a diversity of pro-inflammatory stimuli, as well as on their crosstalk with immune cells (Figure 1, Supplementary Table 1). Collective activities of jakinibs on synovial cellular networks, however, remain to be explored.

### Jakinibs Reduce the Pro-Inflammatory Responses of Synovial Fibroblasts to IL-6 Family of Cytokines

OSM, a member of the IL-6 family of cytokines, has been implicated in the pathogenesis of RA synovitis (44). In synovial fibroblasts, OSM activated the JAK-STAT (45) signaling pathway with early phosphorylation of JAK1 (Y1022/1023), JAK2 (Y1007/1008), JAK3 (Y980), STAT1 (Y701), STAT3 [Y705 (37, 45, 46)] and STAT5 (Y694) (45, 46) and concomitant increase in MCP1 protein secretion, IL-6 mRNA expression and IL-6 protein secretion (37) (Figure 1). Whereas, JAK1 and JAK2 are known to associate with OSM receptor complex, JAK3 interacts specifically with the common γ chain of the receptors for interleukins 2, 4, 7, 9, and 15 (47) but can be phosphorylated also by T-cell receptor independently of γ chain receptors (48). A specific mechanism of the early JAK3 activation in the OSM-stimulated synovial fibroblasts requires further investigation.

Tofacitinib efficiently decreased the OSM-induced production of IL-6 and MCP1, pointing to a pivotal involvement of the JAK-STAT pathway (37, 45) in the expression of these cytokines in synovial fibroblasts (Figure 1). Given the abundancy of IL-6 and MCP1 in RA synovial fluid and the role of synovial fibroblasts in their production (39), jakinibs might importantly diminish the pro-inflammatory cytokine content in synovial fluid in RA. Overall, baricitinib and tofacitinib exhibited similar pan-JAK inhibitory effects in the OSM-stimulated synovial fibroblasts with inhibition of pJAK1-3 and pSTAT1, 3 and 5 (45, 46). Distinct JAKs and STATs, however, exhibited differential baricitinib/tofacitinib sensitivity in the OSM-stimulated synovial fibroblasts. Specifically, a prominent decrease in pJAK3 and pSTAT5 was achieved at 100 nM tofacitinib/baricitinib and of JAK2 at 300 nM tofacitinib/baricitinib, whereas inhibition of pJAK1 and pSTAT3 required high tofactinib/baricitinib doses (500, 1,000 nM) (45, 46). These concentrations were higher than the maximal plasma drug concentrations (<200 nM) in patients with RA, who received therapeutic baricitinib/tofacitinib doses. This implicated that the inhibition of OSM release from immune cells *in vivo* might be required for a complete blockade of STAT3 phosphorylation in synovial fibroblasts.

In contrast to pan-JAK effects of tofacitinib and baricitinib, the JAK3-specific inhibitor PF956980 selectively blocked the OSM-induced pJAK3, pSTAT1, and pSTAT5, but had no effect on pJAK1, pJAK2, and pSTAT3 (46). This suggested that the blockade of pJAK1 and pJAK2 is needed for a successful inhibition of the OSM-induced STAT3 phosphorylation (Figure 1). While tofacitinib and baricitinib decreased the OSM-induced MCP1 and serum amyloid 1, 2 (SAA1, 2) mRNAs in synovial fibroblasts, PF956980 reduced MCP1 only, corroborating the central role of pSTAT3 in the OSM-driven induction of SAA mRNAs in synovial fibroblasts (46). While synovial pJAK3 predominantly localized to CD3+ T cells, vimentin-expressing synovial fibroblasts were also JAK3 positive (46), supporting the role of JAK3 inihbition in synovial fibroblasts in RA.

*IL-6* is a major pro-inflammatory cytokine in the pathogenesis of RA and IL-6 targeting biologicals represent an important pillar in the treatment of RA. Synovial fibroblasts do not express membrane IL-6 receptor but transmit the pro-inflammatory IL-6 signaling primarily via ligation of IL-6 with its soluble IL-6 receptor (sIL-6R), which is abundantly present in RA joints (Figure 1). Stimulation of synovial fibroblasts with IL-6/sIL-6R led to an early phosphorylation of JAK2 (Y1007/8) and STAT3 (Y705) with concomitant increase in SAA1, 2 mRNAs; and tofacitinib (100, 500 nM) efficiently blocked these responses (49). Likewise, JAK2 inhibitor AG490 decreased the IL-6/sIL-6R-induced phosphorylation of STAT3 and the expression of SAA1, 2 mRNA. These experiments pointed to a key role of the IL-6/sIL-6R-JAK2-STAT3 axis in induction of SAA mRNA in synovial fibroblasts (Figure 1).

Like OSM, inhibition of the IL-6-induced pSTAT1, pSTAT3, and MPC1 was dose dependent in synovial fibroblasts and pSTAT1 (IC_50_ = 23 nM) and pSTAT3 (IC_50_ = 77 nM) (50) exhibited differential sensitivity to tofacitinib in synovial fibroblasts. Nonetheless, strong inhibitory effects on IL-6 responses were observed at 100 nM tofacitinib, indicating that the IL-6-driven, and the OSM-induced responses are differentially responsive to JAK inhibition in synovial fibroblasts. Other factors, like the stimulating concentration of IL-6 and OSM, could also contribute to these differential effects. In line with this, 10-fold higher concentrations of tofactinib and baricitinib (1,000, 5,000 nM) were required for decreasing the OSM-induced IL-6 release from synovial fibroblasts when synovial fibroblasts were stimulated with 100 ng/ml of OSM (51), compared to 20 ng/ml OSM (45).

A variety of stimuli, including TNF, can potently induce IL-6 production in synovial fibroblasts, and jakinibs could indirectly interfere with the TNF-induced autocrine IL-6 responses by blocking the IL-6/sIL-6R-JAK1, 2-STAT3 signaling axis (Figure 1).

### Jakinibs Target the TNF-Induced Interferon Responses in Synovial Fibroblasts

TNF plays a principal role in the pathogenesis of RA. Stimulation of synovial fibroblasts with TNF activates signaling through a variety of interconnected intracellular networks, leading to abundant release of pro-inflammatory mediators and matrix remodeling enzymes. Many pathways, including mitogen-activated protein kinase [MAPK – p38, c-JUN] and NF-κB pathways are rapidly activated in the TNF-stimulated synovial fibroblasts (at 1 h after stimulation) (39). The TNF-associated cytokine signature in supernatants of synovial fibroblast cultures significantly correlated with cytokine composition of RA synovial fluid (39), implicating a prominent role of the TNF-stimulated synovial fibroblasts in shaping the pro-inflammatory joint milieu in RA.

In contrast to early activation of the JAK-STAT pathway with IL-6 cytokine family, TNF induced a delayed (at 3–4 h) phosphorylation of STAT1(Y705), but not STAT3 (50, 52), in synovial fibroblasts with accompanying increase in the production of IP-10, MCP1, and RANTES mRNAs and proteins (50) (Figure 1). The TNF-induced gene expression signature in synovial fibroblasts contained more than 50% interferon response genes (53), including many genes with a key role in the pathogenesis of RA [STAT1, CXCL9, CXCL10, B-cell activating factor/tumor necrosis factor superfamily member 13B (BAFF/TNFSF13B)] (Figure 1). Interferon signature genes were shown to be overexpressed in up to 65% patients with RA (54). Additionally, interferon gene signature was associated with an increased risk of developing RA (55) as well as with therapeutic response of RA patients to biological DMARDs (56).

The TNF stimulation of synovial fibroblasts led to an early induction of interferon response factor one (IRF1) protein followed by a delayed phosphorylation of STAT1 (Figure 1). Silencing of IRF1 or inhibition of *de novo* IRF1 synthesis by cycloheximide blocked the phosphorylation of STAT1 and reduced the TNF-driven expression of interferon response genes in synovial fibroblasts, including CXCL9, CXCL10, CXCL11, and BAFF (57). IRF1 was highly expressed in the synovium (54, 57) of RA patients as well as TNF transgenic mice. IRF1 was upregulated in synovial fibroblasts, cultured within the floating Matrigel microspheres, when treated with TNF (57). Overall, these experiments identified TNF as a major driver of synovial IRF1 expression, and the IRF1-pSTAT1 axis as a key regulator of the TNF-induced interferon gene signature in synovial fibroblasts (Figure 1). Specifically, TNF upregulated IRF1, which induced the expression of IFNβ. IFNβ activated STAT1 and the interferon response genes via human type I interferon receptor (IFNAR)-dependent signaling (Figure 1). In line with this, silencing or the antibody-based neutralization of IFNβ significantly impaired the TNF-induced interferon response in synovial fibroblasts (57). Additionally, the silencing of IFNAR in synovial fibroblasts decreased pSTAT1 and reduced the expression of interferon response genes in the TNF-stimulated synovial fibroblasts (57). Neutralizing anti-IFNAR antibodies prevented the TNF-induced production of IP-10 in human synovial fibroblasts and synovial fibroblasts from *ifnar–/–* mice did not upregulate IP-10 in response to TNF (50, 57). Overall, these results provided strong evidence that TNF induces a delayed activation of the JAK-STAT pathway, and turns on interferon response genes in synovial fibroblasts via a secondary, autocrine activation of the IRF1-IFNβ-IFNAR-STAT1 axis (50, 57) (Figure 1).

Baricitinib and tofacitinib (250 nM) suppressed the TNF-induced pSTAT1 and the associated inflammatory interferon response genes (CXCL9, CXCL10, CXCL11, BAFF) in synovial fibroblasts and decreased BAFF expression in synovial fibroblasts within the floating Matrigel microspheres (57). Likewise, 1,000 nM tofacitinib blocked the TNF-induced pSTAT1 in synovial fibroblasts, significantly attenuated the production of MCP1 and RANTES, and neutralized the TNF-driven production in IP-10, but had no effect on IL-8 (50). Overall, this implicated that jakinibs diminish the TNF-driven responses of synovial fibroblasts by interfering with the IFNAR-JAK1/JAK2-driven phosphorylation of STAT1 (Figure 1).

IP-10, RANTES, and MCP-1 were highly abundant in RA synovial fluid, which corroborates the jakinib capacity in diminishing the pro-inflammatory synovial microenvironment in RA joints (39). Additionally, IP-10 promoted the invasiveness of synovial fibroblasts in autocrine and paracrine manner (58) and its inhibition by jakinibs might contribute to beneficial effects of jakinibs on halting joint damage in RA.

### Jakinib Exhibit Limited Effects on Interleukin Beta-Induced Responses of Synovial Fibroblasts

Thousand nanomolar tofacitinib reduced the IL-1β-induced production of IP-10 in synovial fibroblasts (50), while the IL-1β-driven secretion of IL-6, IL-8, and MMP3 was insensitive to tofacitinib and baricitinib even at 5,000 nM concentration (51, 59). In contrast, 5,000 nM peficitinib decreased the IL-1β-induced release of MMP3, MMP1, IL-6, CXCL8, and CXCL1 and 5000 nM filgotinib decreased the IL-1β-stimulated secretion of IL-6 and MCP1 from synovial fibroblasts. Additionally, peficitinib (1,000, 5,000 nM) blocked basal migration of synovial fibroblasts, while peficitinib and baricitinib (5,000 nM) inhibited the IL-1β-induced proliferation of synovial fibroblasts. Overall, high doses of jakinibs were required for the interference with IL-1β responses in synovial fibroblasts, suggesting a JAK-STAT-independent mechanism of action, e.g., by affecting other kinases. This also inferred that high amounts of IL-1β in the inflamed RA joints might contribute to unresponsiveness to jakinibs at their therapeutic *in vivo* concentrations. These effects could be less pronounced with filgotinib (C_max_ 3,360 nM at 200 mg once daily) (60) which achieves a higher plasma concentration when administered in therapeutic dose.

### Jakinibs Impair Responses of Synovial Fibroblasts to IFNγ

IFNγ increased migration and invasion of synovial fibroblasts, accompanied with an early induction of pSTAT1 (Y701) and an increase in phosphorylation of focal adhesion kinase (FAK) at Y925 (61) (Figure 1). FAK controls cellular migration by regulating the turnover of focal adhesions (62). Silencing of JAK2 but not JAK1 in synovial fibroblasts diminished phosphorylated focal adhesion kinase (pFAK Y925) amounts (61). High dose baricitinib (1,000 nM, 5,000 nM) abrogated the IFNγ-induced pSTAT1 (Y701), decreased pFAK(Y925) and attenuated the invasion of IFNγ-stimulated synovial fibroblasts (61). These experiments suggested a potential regulatory effect of baricitinib on the crosstalk between T cells and synovial fibroblasts via blocking the IFNγ-JAK2-pFAKY925 signaling cascade in synovial fibroblasts. Yet, high baricitinib concentrations were needed for these effects, which are not reached in RA patients treated with baricitinib. This indicated that blocking IFNγ release from T cells could support the jakinib-driven inhibition of IFNγ responses in synovial fibroblasts.

### Jakinibs Influence Pro-Inflammatory Outputs of IL-17-Induced Pathways in Synovial Fibroblasts

Treatment of synovial fibroblasts from RA patients with IL-17 increased phosphorylation of STAT3, which was accompanied with enhanced production of IL-6 (37). Tofacitinib (1,000 nM) inhibited the IL-17-induced production of IL-6 in synovial fibroblasts (Supplementary Table 1) (37). Since IL-17 does not directly activate the JAK-STAT pathway, the mechanism of the IL-17 driven STAT3 phosphorylation needs to be elucidated. Several IL-17-induced pathways (63), including AP-1 and NF-κB signaling are coupled to the JAK-STAT signaling. Inhibition of these indirect responses may contribute to the observed effects of tofacitinib in the IL-17-stimulated synovial fibroblasts.

### Jakinibs Alter Synovial Bioenergetics in Synovial Explants and Synovial Fibroblasts

Under pro-inflammatory and hypoxic condition synovial fibroblasts switch from mitochondrial to glycolytic production of ATP (37). Consequently, lactate and succinate accumulate in RA synovial fluid and tissue (64–67). Increased amounts of succinate induced the release of IL-1β from macrophages (68). Meanwhile, the IL-1β-induced responses of synovial fibroblasts were rather resistant to JAK inhibition (51), suggesting that modulating the metabolism may facilitate the Jakinib-driven inhibition of pro-inflammatory and matrix-degrading responses of synovial fibroblasts.

Tofacitinib significantly modulated synovial bioenergetics and decreased glycolytic environment in synovial fibroblasts, which might potentiate its anti-arthritic effects (37). Specifically, tofacitinib (1,000 nM) decreased mitochondrial membrane potential, mitochondrial mass and reactive oxygen species (ROS) production and regulated key mitochondrial genes (37) in synovial fibroblasts from patients with RA. Oxidative phosphorylation, production of ATP, the maximal respiratory capacity, and the respiratory reserve significantly increased in the tofacitinib-treated synovial fibroblasts, while glycolysis and the expression of key glycolytic enzymes (hexokinase 2, glycogen synthase 3 alpha, lactate dehydrogenase A and HIF-1α) were suppressed. Similar changes in metabolic gene expression were observed in RA synovial explants treated with tofacitinib (37). Additionally, tofacitinib reversed the OSM-induced metabolic switch in synovial fibroblasts, shifting the metabolism away from glycolysis (37). This positioned the JAK-STAT signaling and its inhibition with jakinibs at the crossroad of synovial metabolism and inflammation in RA.

### Jakinib Effects on Fibroblast-Immune Cell Crosstalk

Intracellular signaling pathways are highly interconnected. A systemic exploration and deeper understanding of crosstalk between the JAK-STAT and other signaling pathways might uncover synergistic therapeutic mechanisms of jakinibs. Additionally, this could broaden the understanding of jakinib adverse effects and foster drug discovery in RA. We have highlighted above some of the JAK-STAT interacting hubs in synovial fibroblasts, including TNF-IRF1-IFNβ-IFNAR-STAT1 (50, 57) and IFNγ-JAK2-pFAKY925 (61) pathways. Jakinibs blocked these pathways in synovial fibroblasts, albeit with a different sensitivity. Targeting these pathways could efficiently break vicious cycle of the stromal-immune cell interactions in RA synovium. Specifically, suppression of the TNF-IRF1-IFNβ-induced secretion of IP-10 and BAFF by tofacitinib might decrease the IP-10-driven recruitment of T cells into the inflamed RA synovium and the BAFF-dependent proliferation, differentiation and antibody production by B cells. Neutralization of IP-10 effects with neutralizing antibodies inhibited inflammation and bone destruction *in vivo* in experimental models of RA (69, 70). Similar to synovial fibroblasts, macrophages, and endothelial cells activated the TNF-IRF-IFNβ-pSTAT1 inflammatory axis (71, 72). This suggests that jakinibis might exhibit their anti-arthritic actions across diverse synovial cell types and pathotypes in RA, which could contribute to their efficacy in RA.

### Jakinibs Interfere With Non-JAK-STAT Signaling Pathways in Synovial Fibroblasts

IL-6 and IFNγ predominantly activate the JAK-STAT pathway; MAPK signaling, however, can also be activated in the presence of IL-6 and IFNγ (73). OSM increased phosphorylation of different components in the MAPK pathway in synovial fibroblasts, including extracellular regulated kinase (ERK), p38 and c-JUN N-terminal kinase 1/2 (JNK1/2) (45). Tofacitinib attenuated these OSM-induced effects (45). Additionally, treatment with p38 inhibitor resulted in almost complete inhibition of the OSM-induced IL-6 release, pointing toward an interplay between the JAK-STAT and MAPK pathways in synovial fibroblasts.

In summary, jakinibs inhibited the JAK-STAT signaling in synovial fibroblasts under various pro-inflammatory stimuli *in vitro* by blocking the early as well as the delayed, secondary activation of the JAK-STAT signaling. This enabled suppression of a broad variety of cytokine-induced responses in synovial fibroblasts *in vitro* which could terminate the cytokine-driven vicious cycles between synovial fibroblasts and immune cells. In synovial fibroblasts, jakinibs diminished the secretion of the major pro-inflammatory components of RA synovial fluid, but not IL-8. Contrarily, jakinibs effectively suppressed IL-8 release from synovial explants, indicating the primary role for immune cell effects in the jakinib-driven IL-8 attenuation. Jakinib effects were context-dependent in synovial fibroblasts, as reflected in their variable potency under different pro-inflammatory stimuli. This might define the interpatient variability in responding to jakinibs in RA. Additionally, jakinib-dependent suppression of cytokine release from immune and inflammatory cells might be required to curb pathogenic fibroblast activities *in vivo*, particularly in settings where jakinib doses exceeded therapeutic window.

## *In vivo* Effects of Jakinibs in Experimental Models of RA

Rabbits with antigen-induced arthritis (AIA) were treated with tofacitinib (10 mg/kg/day), starting at the second week after induction of AIA. In this regimen, tofacitinib significantly decreased global synovitis score (Krenn score: inflammatory cell infiltration and stromal hypertrophy decreased) but did not intervene with systemic inflammation (as measured by C reactive protein) 4 weeks after initial antigen injection. Tofacitinib normalized the expression of Tnf, Il-6, and Ifnγ mRNAs, decreased Mmp1, 3, and 13 mRNAs but did not diminish increased Il-1β mRNA in synovial tissue. These effects were accompanied by the downregulation of synovial pStat1, whereas pStat3 and NF-κB activities were not diminished. Tofacitinib differentially affected intrinsic inhibitors of the Jak-Stat signaling, decreasing synovial Socs1, but not synovial Socs3 protein. This suggested that tofacitinib reduces chronic synovitis in AIA primarily via blockade of pStat1.

In AIA rat model, tofacitinib (74) decreased the histopathological inflammation score and Il-6 mRNA expression. Tofacitinib suppressed the osteoclast-mediated bone resorption by reducing synovial receptor activator of NF-κB Ligand (Rankl) expression and production of Rankl by human T lymphocytes. Likewise, tofacitinib (75) reduced joint inflammation (reduced inflammatory cell infiltration), decreased cartilage damage and bone erosions, while increasing cortical and trabecular bone hardness. This was accompanied with reduced pStat1 and Socs1 protein amounts in bone of AIA rats. The amounts of Il-6, Il-17, Rankl, and osteoprotegerin (but not Tnf, Il-1β) proteins were attenuated and bone turnover markers (carboxy-terminal telopeptide of type I collagen and procollagen type I propeptides) were diminished in serum of AIA rats.

In mice with collagen induced arthritis (CIA), tofacitinib (76) significantly improved arthritis score, inhibited osteoclastogenesis and decreased joint destruction. In this model, synovial tissues exhibited increased pStat3, but not Stat1 and Stat5. Tofacitinib efficiently decreased pStat3 in mouse synovial joints with concomitant reduction in synovial Il-6 and Rankl mRNAs and serum Il-6 protein. This indicated that tofacitinib has beneficial effects on inflammation as well as joint destruction in CIA via blockade of the Stat3-Il-6-Rankl axis.

The therapeutic effects of upadacitinib and tofacitinib were compared in AIA rats (77). Rats were treated between day 7 and 17 after induction of AIA and drugs were administered twice daily. Upadacitinib decreased synovial inflammation, synovial hypertrophy, cartilage damage and bone erosion. Tofacitinib was similarly effective; however, the total efficacious exposure to drug was much smaller for upadacitinib compared to tofacitinib. This increased *in vivo* potency of upadacitinib could reflect an increased cellular potency of upadacitinib for Jak1 compared to tofacitinib. In naïve rats, 14 day-tofacitinib, but not upadacitinib, suppressed natural killer (NK) cell counts and reticulocyte deployment. This suggested that at *in vivo* therapeutic doses, upadacitinib exhibits an improved benefit: risk profile, highlighting the role of Jak3 inhibition in the tofacitinib-induced side effects (74, 75). Several other *in vivo* models (78) showed beneficial anti-arthritic effects of different jakinibs, including baricitinib (79), peficitinib (80), ruxolitinib (81), filgotinib (82), and decernotinib (83).

Dual kinase inhibition with tofacitinib and spleen tyrosine kinase (Syk) inhibitor PRT062607 was shown as advantageous strategy when compared to a single kinase inhibition in preventing as well as ameliorating chronic G6PI-induced arthritis (84). Dual kinase inhibition blocked several arthritis-relevant pathways and cell functions, specifically Th1/Th17 cytokine cascade, osteoclastogenesis and invasiveness of synovial fibroblasts.

Results from experimental *in vivo* models are in line with the observed efficacy of jakinibs in patients with RA. These models provided important insights into mechanistic role of the activated Jak-Stat pathways in inflammation and joint damage in arthritis *in vivo*. Additionally, new strategies for therapeutic targeting of arthritis have been opened as exemplified by dual kinase targeting approach (84).

## Conclusions

In this review, we explored the effects of jakinibs on synovial pathology in inflammatory arthritis, primarily focusing on jakinib actions on synovial fibroblast-dependent disease mechanisms. We compiled published data from RA clinical trials, experimental *in vivo* models as well as *ex vivo* and *in vitro* studies on human synovial explants and cultured human synovial fibroblasts. We discussed the effects of tofacitinib on halting structural joint damage and synovitis in RA clinical trials. By summarizing experimental findings from animal models of RA, we further delved into molecular mechanisms and therapeutic benefits of jakinibs on synovial pathology *in vivo*. Finally, we highlighted the cytokine-based activation and the jakinib-driven inhibition of the JAK-STAT pathways in cultured synovial fibroblasts under diverse experimental designs and conditions. We stressed the pathways, through which jakinibs could interfere with the crosstalk between synovial fibroblasts and immune cells, thereby terminating vicious cycles of joint inflammation and damage in RA. Overall, this review highlights the current knowledge on jakinib effects on synovial pathology, challenges current experimental designs and opens new perspectives for jakinib discovery and development in future.

## Author Contributions

BB and MF-B critically evaluated the literature and wrote the paper. TM reviewed the clinical trials and co-wrote the paper.

## Conflict of Interest

The authors declare that the research was conducted in the absence of any commercial or financial relationships that could be construed as a potential conflict of interest.
